# Person-centered evaluation of positive body image among Chinese nursing students: Associations with body appreciation, self-concept clarity, and negative affect

**DOI:** 10.3389/fpsyg.2022.1088702

**Published:** 2023-01-06

**Authors:** Ting Zhang, Dan Su, Shuwen Li, Yajuan Yang, Huiping Li

**Affiliations:** School of Nursing, Anhui Medical University, Hefei, China

**Keywords:** latent profile analysis, self-concept, body appreciation, negative affect, students

## Abstract

**Background:**

Nowadays, the effect of body image (BI) on negative affect among nursing college students has been ignored. This study used person-centered methods to evaluate the profiles of body appreciation (BA) among Chinese nursing students. We explored the predictive factors of these profiles and examined the difference in negative affect among the profiles.

**Methods:**

A cross-sectional survey study was conducted in a university in Anhui province, China. Five hundred and forty-one Chinese nursing students from a nursing college of a larger university completed this survey. Participants completed the Sociodemographic Characteristics Questionnaire, the Self-concept Clarity Scale, the Body Appreciation Scale-2, and the Depression Anxiety Stress Scale. Latent profile analysis was employed to classify the BA profiles, and multinomial logistic regression analysis were conducted using the three-step regression and Bolck-Croon- Hagenaars method to test the relationship between the latent profiles of BA, several covariates (including gender, age, year of schooling, residence, academic achievement level, interpersonal relationship status, whether the student is an only child, duration of use of social media platforms, body mass index (BMI), and self-concept clarity (SCC)), and negative affect.

**Results:**

Analysis resulted in a four-profile model that included a “low BA” group (14.42%), an “upper medium BA” group (36.41%), a “lower medium BA” group (36.97%), and a “high BA” group (12.20%). SCC, years of schooling, interpersonal relationship status, and BMI were found to be the predictive factors of BA profiles. The four profiles differed in levels of stress, depression, and anxiety.

**Conclusion:**

Our study showed that more than half of nursing students were dissatisfied with their BI and the predictive factors evaluated and negative affect were associated with the profiles of BA. Nursing faculties should take measures to help students develop a more appropriate awareness of nursing aesthetics, a healthier lifestyle, a better interpersonal relationship with family members and peers, and a clearer self-concept to promote body appreciation and reduce negative affect related to BI.

## Introduction

Nowadays, the emergence of social media has facilitated a dramatic growth in the quantity and speed of distribution of social information. However, the drawbacks of this process are becoming evident. Negative social media exposure has been shown to have harmful influence on the body image (BI), emotions, and living habits of young women (Rounsefell et al., 2020; Ouyang et al., 2021). BI has been widely treated as a complex construct consisting of personal perception, body-related cognition, attitudes, behavior, and affect toward one’s own physical appearance (Jarry et al., 2019). BI has attracted much attention worldwide, and researchers have affirmed its close association with mental and physical health (Lemes et al., 2018). Moreover, many research have proven that females report worse BI than males across their lifespan, and this disparity has an important impact on their quality of life (da Silva et al., 2020). The majority of nurses and nursing college students are female. They have experienced high level of pressure for a perfect BI (King et al., 2009; Duarte et al., 2021). However, in existing nursing-education-related research, researchers have concentrated on students’ unpleasant emotions related to academic training or clinical practice (Tung et al., 2018). The effect of other unique factors such as BI on negative affect has been ignored.

Extant research has tended to use body satisfaction or dissatisfaction as a measure of an individual’ s BI (Aparicio-Martinez et al., 2019; Blum et al., 2021). Most research have specifically focused on negative BI (Aparicio-Martinez et al., 2019). Negative BI is widespread among female, and has been linked to a range of health problems (Lemes et al., 2018). Moreover, it has been shown that negative BI can be affected by physical, sociodemographic, intrapersonal and interpersonal variables, such as age, sex, internalization of the thin ideal, self-objectification, daily communication with peers and family members, media use, and sociocultural influence (Barbeau et al., 2022). According to the Tripartite Influence Model, social influence from media, parents, and peers contribute to the development and maintenance of negative BI (Aparicio-Martinez et al., 2019). This sociocultural model also recommends that the association between social influences and BI is mediated by both internalization of the thin ideal and social comparisons (Aparicio-Martinez et al., 2019). Then some researchers examined and extended this sociocultural model among college students. They confirmed the key role of self-concept clarity (SCC) in the formation of negative BI. SCC is defined as “the extent to which the contents of the self-concept are clearly and confidently defined, internally consistent, and temporally stable” (Na et al., 2018). Vartanian and Dey (2013) found that thin-ideal internalization and social comparisons mediated the relationship between SCC and negative BI among female university students from Australia. Seo et al. (2020) tested the mediational role of SCC in the influence of parents and media on BI and depression among Korean college students. To summarize the research, low SCC can lead to negative BI.

Self-view is an integral part of the self and is a complex, multidimensional concept (Na et al., 2018). Self-concept, a cognitive generalization of one’ s self-view based on past experiences (Na et al., 2018). It has been well demonstrated that the self-concept was dynamic, especially in response to changes in the social environment (Na et al., 2018). SCC represents the ability to establish a consistent and stable sense of self (Na et al., 2018). For female college students, social media use is the most important influence on BI (Aparicio-Martinez et al., 2019). When students lack a clear sense of their own identity, they are more likely influenced by the media (Vartanian and Dey, 2013). They may highly value others’ perception of their external appearance, and regard different kinds of web celebrities or stars as their beauty ideals, adopting their style of dress and even attempting to perfect their appearance through unhealthy habits (Luo et al., 2019). Over time, women who internalize the thin ideal and engage in social comparison are more likely to experience greater pressure for negative BI and suffer from negative affect as a result (Griffiths et al., 2018).

Because BI is a multidimensional construct, and owing to the emergence of the field of positive psychology, positive BI has been increasingly favored by researchers in recent years (Tiggemann and Hage, 2019). Being an important element of positive BI, body appreciation (BA) is a complicated construct that involves not just satisfaction with one’ s BI, but also essential characteristics (Turan et al., 2019; Tiggemann and Hage, 2019). Recent research showed a significant association between BA and mental health among women (Geller et al., 2022). In another study, BA had a negative influence on depressive symptoms among a diverse sample of college students (Liu et al., 2022). In one study, the relationship between BA and health-related quality of life was moderated by age and, in particular, was negatively associated with health-related quality of life in women younger than 36 years old (O’Neill et al., 2018). Other studies confirmed that younger people experienced lower mental well-being and BA compared to older people (Trindade et al., 2018). Collectively, while studies on BA are still limited, existing findings indicate that BA has a strong influence on the psychological health of women, especially young women. As mentioned above, the development and evaluation of positive BI are not simply the inverse of negative BI (Turan et al., 2019). And the predictors of positive BI are not opposite of predictors of negative BI (Turan et al., 2019). However, only a handful of studies have examined predictors of BA. It has been clarified that physical (body mass index (BMI)), sociodemographic (gender, age), interpersonal (appearance media, caregiver eating messages, adult attachment) and intrapersonal (gratitude, perfectionism, self-objectification, contingent self-worth, social comparison, thin-ideal internalisation) characteristics promoted the development of BA (Babayan et al., 2018; Baceviciene and Jankauskiene, 2020; Gui et al., 2020; Yamamiya et al., 2021). However, the predictive effect of SCC on BA has not been examined. Besides, BI is influenced by sociocultural factors (Turan et al., 2019). However, in non-Western contexts such as China, the evaluation of BA among college students has been limited.

Nursing profession not only deals with individual’ s physical health but helps in promoting their quality of life. However, such a respected group representing the role models to the public yet also to be negative with their BI (Duarte et al., 2021). It seems that nurses with negative BI are more likely to adopt disordered eating behaviours, which in turn worsen their physical and mental health (King et al., 2009). Moreover, poor physical and mental health of nurses may adversely affect their work engagement, as a consequence of decreasing their quality of nursing care services (Zhang et al., 2021). A further concern is that nurses’ attitudes toward the patients may be negatively influenced by their own negative BI (Goad et al., 2018). That is, the BI of the patients they will care for may be affected by the patient’ s illness or treatment, and their negative attitudes toward BI promotes avoidance of patients, resulting in the contradicting the nursing profession mission (Goad et al., 2018; Duarte et al., 2021). More importantly, when nurses place their personal prejudice on patients, their willingness to provide support maybe negatively affected (Ak et al., 2021). Additionally, patients’ uncomfortable experiences of nursing care often lead to decrease willingness to search for medical services, resulting in producing unsatisfied treatment outcomes (Ak et al., 2021). Incorporating BA within the nursing may help nurses and nursing students appreciate, respect, and accept their BI and enable them to form positive attitudes toward the patients’ BI and their nursing care (Goad et al., 2018). However, relevant research focused on nursing students has so far been limited.

An drawback of most current research related to BI is that it uses a variable-centered approach. While this approach can explore the important relationship between BI and certain variables, and enables generalized conclusions across samples, variable-centered design overlooks differences within individuals (Shin et al., 2019). In contrast, person-centered approaches consider population diversity, explore distinct potential subpopulations, and confirm variable associations at the intrapersonal level (Cowden et al., 2021). Because BI is a multidimensional concept, affected by both internal and external factors (Jarry et al., 2019; Barbeau et al., 2022), a person-centered approach may be a more effective method of studying it. For example, different types of BI may be prevalent among different populations (Jarry et al., 2019). Population diversity need be studied to bring the results more in line with the characteristics of samples, and to enable more nuanced targeted interventions in the long run. Person-centered research of BI among Chinese college students is limited.

Based on the preceding overview, our research aimed to evaluate the profiles of BA among Chinese nursing students. We also explored the predictive factors of these profiles and examined the difference in negative affect among the profiles. Accordingly, first, we used Latent profile analysis (LPA) to determine the potential profiles of BA among Chinese nursing students, a person-centered approach using continuous data that constructs a typology or clustering to classify observational units into a set of latent classes (Weller et al., 2020). LPA can also be used to reduce numerous categorical variables or continuous variables to a few subgroups and explore the relationships between those variables and others (Weller et al., 2020). Second, we used the results of LPA to explore the predictive factors of these profiles. As aforementioned, BI is socially determined. Thus we selected gender, age, whether the student is an only child, year of schooling, residence, and academic achievement level as sociodemographic predictive factors that represent students’ characteristic of social context for the profiles of BA. And based on the Tripartite Influence Model, interpersonal (interpersonal relationship status, duration of use of social media platforms) and intrapersonal (SCC) variables should not be ignored. Furthermore, prior studies have examined the relationship between BA and BMI, but the results are controversial (He et al., 2020). Recently, a meta-analysis showed that the overall negative relationship between BA and BMI was weak among males, but slightly larger among females (He et al., 2020). So we treated it as a potential physical predictive factor for the profiles of BA. Third, we verified the relationship between such profiles and negative affect. This study can provide baseline data for designing educational programs to promote the development of positive BI and coping with negative affect among Chinese nursing students. And the findings of our study will provide evidence for developing interventions to improve negative BI among non-Chinese students as well.

## Materials and methods

### Study setting and participants

We conducted a cross-sectional survey from January to May of 2021 in a nursing college of a university in Anhui Province, China, using convenience sampling. To meet inclusion criteria, participants needed to have passed the national college entrance examination and be full-time students who volunteered for the study. Exclusion criteria included psychiatric disorders and refusal to participate. We used a cross-sectional sample equation to estimate the sample size, *n* = μ_α/2_^2^ S^2^/δ^2^, where μ_α/2_ represents the value of a two-tailed level test (α = 0.05, μ_α/2_ = 1.96), S is the standard deviation taken from a normal distribution from our pilot study in 40 nursing students (S = 0.978, the standard deviation score of BA), and δ is the allowed error (δ = 0.10 in this study). Considering the 20% non-response rate, 461 students were recruited to ensure an adequate basis for analysis. A total of 592 questionnaires were collected, 541 of which were valid and 51 were excluded due to the regular answer and incomplete questionnaires, resulting in an 91.39% effectiveness rating.

### Ethical consideration

After approval by the ethics committee of the university (Approval No. 20210075), we explained the related information about the research to all students. Consent forms were also received before they filled in the questionnaires.

### Measurement

The self-report questionnaire included the Sociodemographic Characteristics Questionnaire (SCQ), the Self-concept Clarity Scale (SCCS), the Body Appreciation Scale-2 (BAS-2), and the Depression Anxiety Stress Scale (DASS-21). And we have received the permission to use these scales.

### Sociodemographic characteristics

The SCQ was used to collect the demographic characteristics and academic-related data of the students. Items on the questionnaire include gender, age, grade, residence, whether the student is an only child, and duration of use of social media platforms.

### SCC

To assess the extent of the individual’ s SCC, we used the SCSS (Campbell et al., 1996). This unifactorial scale includes 12 items (e.g., “In general, I have a clear sense of who I am and what I am.”) which are rated from 1 (strongly disagree) to 5 (strongly agree; Campbell et al., 1996). The Chinese version of SCCS was previously verified as having adequate internal consistency (Liang et al., 2022). The internal consistency coefficients was 0.815 (Liang et al., 2022). In our sample, Cronbach’s alpha coefficients for the total scale was 0.77.

### BA

The BAS-2 was developed to measure the degree of an individual’ s BA (Tylka and Wood-Barcalow, 2015). The Chinese version is a one-dimensional scale, which has been verified using confirmatory factor analysis (Swami et al., 2016). The scale consists of 10 items (e.g., “I appreciate the different and unique characteristics of my body.”), with each item scored from 1 (completely disagree) to 5 (completely agree; Swami et al., 2016). CFA was performed to assess the 10-item single factor model, and the model adequately fit the data, RMSEA = 0.078, χ^2^ (24) = 7.72, CFI = 0.967, TLI = 0.937, SRMR = 0.030 (Swami et al., 2016). The internal consistency coefficients for women and men were 0.89 and 0.86, respectively (Swami et al., 2016). In the current study, Cronbach’s alpha coefficient of the whole scale was 0.89.

### Depression, anxiety, and stress

The Depression Anxiety Stress Scale (DASS-21) was used to measure the individual’ s state as an indirect assessment of negative affect over the past week (Parkitny, 2010). The scale contains subscales for depression, anxiety, and stress, each with seven items (Parkitny, 2010). Each item is rated from 0 (did not apply to me at all) to 3 (applied to me very much; Parkitny, 2010). The internal consistency index of the Chinese version was 0.956 for the total scale (Chen et al., 2022). In this study, the Cronbach’s alpha coefficient of the depression, anxiety, and stress subscales, as well as the entire scale, was 0.92, 0.90, 0.89, and 0.97, respectively.

### Data collection procedure

With approval from the ethics committee of the university (Approval No. 20210075), two trained researchers explained the purpose, importance, and methods of the study to all participates. Students first filled out consent forms and then were given standardized instructions prior to filling out the questionnaires independently. Students took about 10 min to finish the questionnaires before starting their classes.

### Data analysis

We analyzed the data using IBM SPSS statistics version 20.0 for Windows (IBM Corp., Armonk, NY, United States) and Mplus 7.4. We used LPA to determine the potential profiles of BA among Chinese nursing students. The acceptable criteria for fit statistics are as follows: 1) lower Akaike information criterion (AIC), Bayesian information criterion (BIC), and sample-size-adjusted BIC (a-BIC) values; 2) the entropy test with values greater than 0.80, examining more accurate classification; and (3) significant value of p on the Lo–Mendell–Rubin likelihood ratio test (LMR-LRT) and Bootstrapped likelihood ratio test (BLRT; Weller et al., 2020). The following were used as predictors of different profiles of BA: gender, age, year of schooling, residence, academic achievement level, interpersonal relationship status, whether the student is an only child, duration of use of social media platforms, age at which student began to use the Internet, BMI, and SCC. These covariates of profile groups were tested by the three-step regression (R3STEP; Weller et al., 2020). Shin et al. (2019) found that Bolck-Croon-Hagenaars (BCH) method was the most robust for dealing with auxiliary distal outcomes, compared with three-step maximum likelihood and Lanza-Tan-Bray approaches. So the distal outcome variable was tested using the BCH method to explore the relationship between the latent profiles and negative affect (Shin et al., 2019). *p* values <0.05 were considered statistically significant.

## Results

### Descriptive analysis

Students’ ages ranged from 17 to 25, with a median of 20 and a quartile spacing of 3, most of them were female (*n* = 391; 72.3%). As for year of schooling, 234 (43.3%) participants were freshman-level nursing students, 196 (36.2%) participants were sophomore-level nursing students, 55 (10.2%) participants were junior-level nursing students, and 56 (10.4%) participants were senior-level nursing students. In terms of residence, 120 (22.2%) students came from urban areas, 421 (77.8%) students were from rural areas. Demographic data of Chinese nursing students are shown in Table 1.

**Table 1:** Comparison of demographic information among four body appreciation profiles (N = 541).

Category	Subcategory	Total sample	Class 1	Class 2	Class 3	Class 4	χ^2^	*p*
Gender, n (%)	Male	150 (27.7)	23 (29.5)	55 (27.9)	52 (26.0)	20 (30.3)	0.640	0.887
	Female	391 (72.3)	55 (70.5)	142 (72.1)	148 (74.0)	46 (69.7)		
Age, n (%)	<18 years old	15 (2.8)	2 (2.6)	9 (4.6)	3 (1.5)	1 (1.5)	4.559	0.601
	18–20 years old	365 (67.5)	50 (64.1)	131 (66.5)	139 (69.5)	45 (68.2)		
	≥21 years old	161 (29.8)	26 (33.3)	57 (28.9)	58 (29.0)	20 (30.3)		
Year of schooling, n (%)	Freshman	234 (43.3)	31 (39.7)	86 (43.7)	86 (43.0)	31 (47.0)	19.799	0.019
	Sophomore	196 (36.2)	29 (37.2)	75 (38.1)	79 (39.5)	13 (19.7)		
	Junior	55 (10.2)	10 (12.8)	16 (8.1)	14 (7.0)	15 (22.7)		
	Senior	56 (10.4)	8 (10.3)	20 (10.2)	21 (10.5)	7 (10.6)		
Residence, n (%)	Urban	120 (22.2)	20 (25.6)	36 (18.3)	42 (21.0)	22 (33.3)	7.200	0.066
	Rural	421 (77.8)	58 (74.4)	161 (81.7)	158 (79.0)	44 (66.7)		
Academic achievement level, n (%)	Good	140 (25.9)	19 (24.4)	63 (32.0)	39 (19.5)	19 (28.8)	14.100	0.029
	medium	364 (67.3)	49 (62.8)	125 (63.5)	146 (73.0)	44 (66.7)		
	Poor	37 (6.8)	10 (12.8)	9 (4.6)	15 (7.5)	3 (4.5)		
Interpersonal relationship status, n (%)	Good	284 (52.5)	31 (39.7)	117 (59.4)	89 (44.5)	47 (71.2)	23.240	0.000
	Poor	257 (47.5)	47 (60.3)	80 (40.6)	111 (55.5)	19 (28.8)		
Being an only child, n (%)	Yes	185 (34.2)	33 (42.3)	63 (32.0)	65 (32.5)	24 (36.4)	3.104	0.376
	No	356 (65.8)	45 (57.7)	134 (68.0)	135 (67.5)	42 (63.6)		
Duration of use of social media platforms, n (%)	<30 min	19 (3.5)	2 (2.6)	5 (2.5)	9 (4.5)	3 (4.5)	19.461	0.078
	30 min-1 h	72 (13.3)	10 (12.8)	35 (17.8)	20 (10.0)	7 (10.6)		
	1 h-2 h	108 (20.0)	11 (14.1)	50 (25.4)	34 (17.0)	13 (19.7)		
	2 h-3 h	98 (18.1)	16 (20.5)	38 (19.3)	33 (16.5)	11 (16.7)		
	>3 h	244 (45.1)	39 (50)	69 (35.0)	104 (52.0)	32 (48.5)		
BMI, n (%)	Underweight	108 (20.0)	12 (15.4)	45 (22.8)	33 (16.5)	18 (27.3)	12.748	0.047
	Normal	366 (67.7)	53 (67.9)	136 (69.0)	134 (67.0)	43 (65.2)		
	Overweight/obese	67 (12.4)	13 (16.7)	16 (8.1)	33 (16.5)	5 (7.6)		

Class 1, “low BA” group; Class 2, “upper medium BA” group; Class 3, “lower medium BA” group; Class 4, “high BA” group.

According to the results of one-sample Kolmogorov–Smirnov tests, the scores of SCSS and BAS-2 should be expressed by the “mean (standard deviation)” with normality distribution (*p* > 0.05), while the scores of the three subscales of DASS-21 were expressed as “median (interquartile range [IQR])” due to the skewed distribution (*p* < 0.01). So the average scores of body appreciation, self-concept clarity, depression, anxiety, and stress among nursing students were 34.61 (7.65), 39.56 (6.91), 10 (14), 12 (12), and 14 (12) respectively.

### Correlation analysis

Results of the correlation analysis are shown in Table 2. The stress, anxiety, and depression of nursing students were significantly and negatively correlated with BA (*p* < 0.01) and SCC (*p* < 0.01). SCC was significantly and positively correlated with BA (*p* < 0.01).

**Table 2:** Correlations of variables.

Variables	1	2	3	4	5
1. Body appreciation	1.000				
2. Self-concept clarity	0.194**	1.000			
3. Stress	−0.175**	−0.220**	1.000		
4. Anxiety	−0.154**	−0.190**	0.886**	1.000	
5. Depression	−0.175**	−0.161**	0.863**	0.883**	1.000

**p < 0.01.

### Latent profile analysis

**Figure 1:**
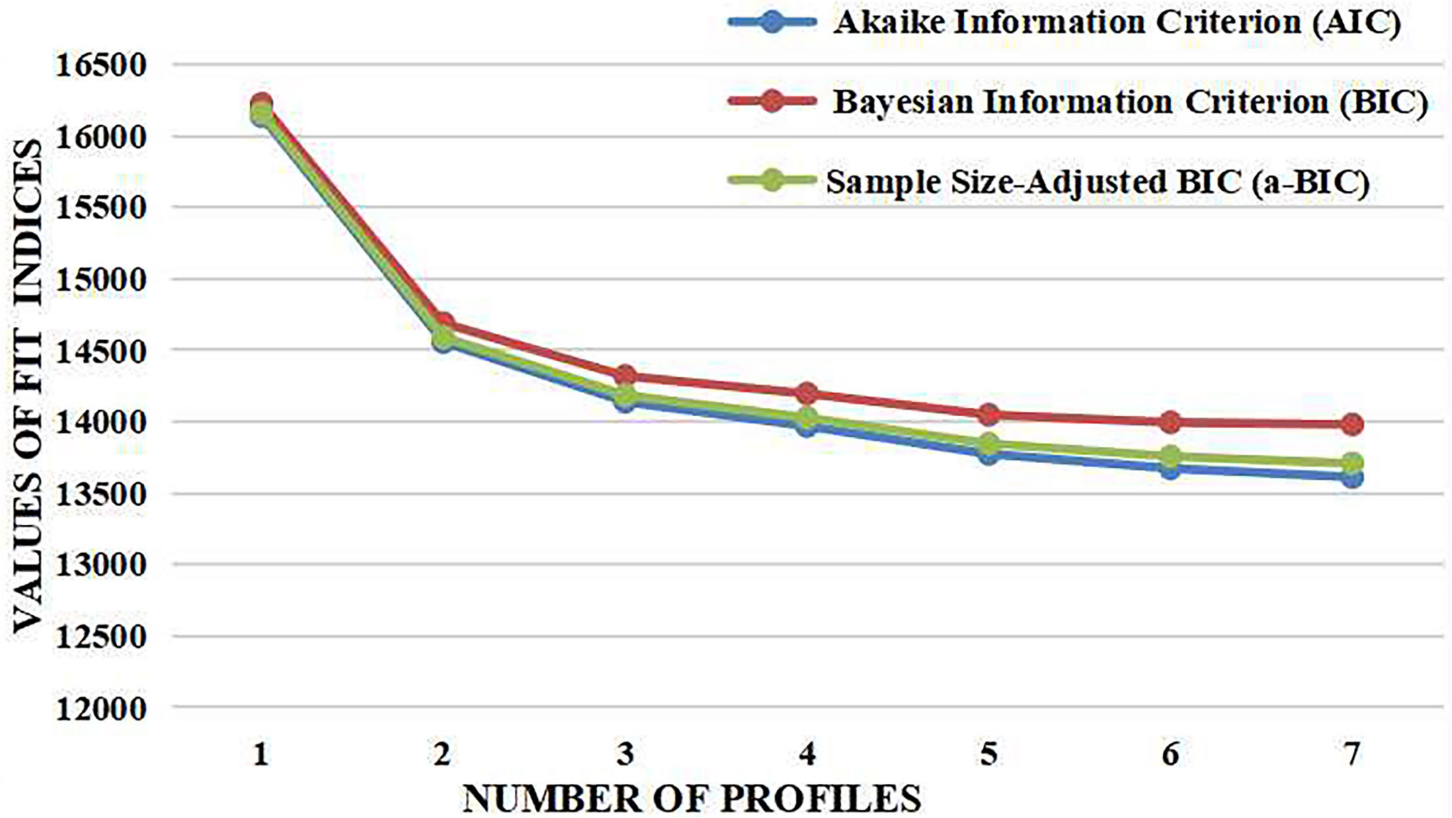
Elbow plot for the fit indices.

**Figure 2:**
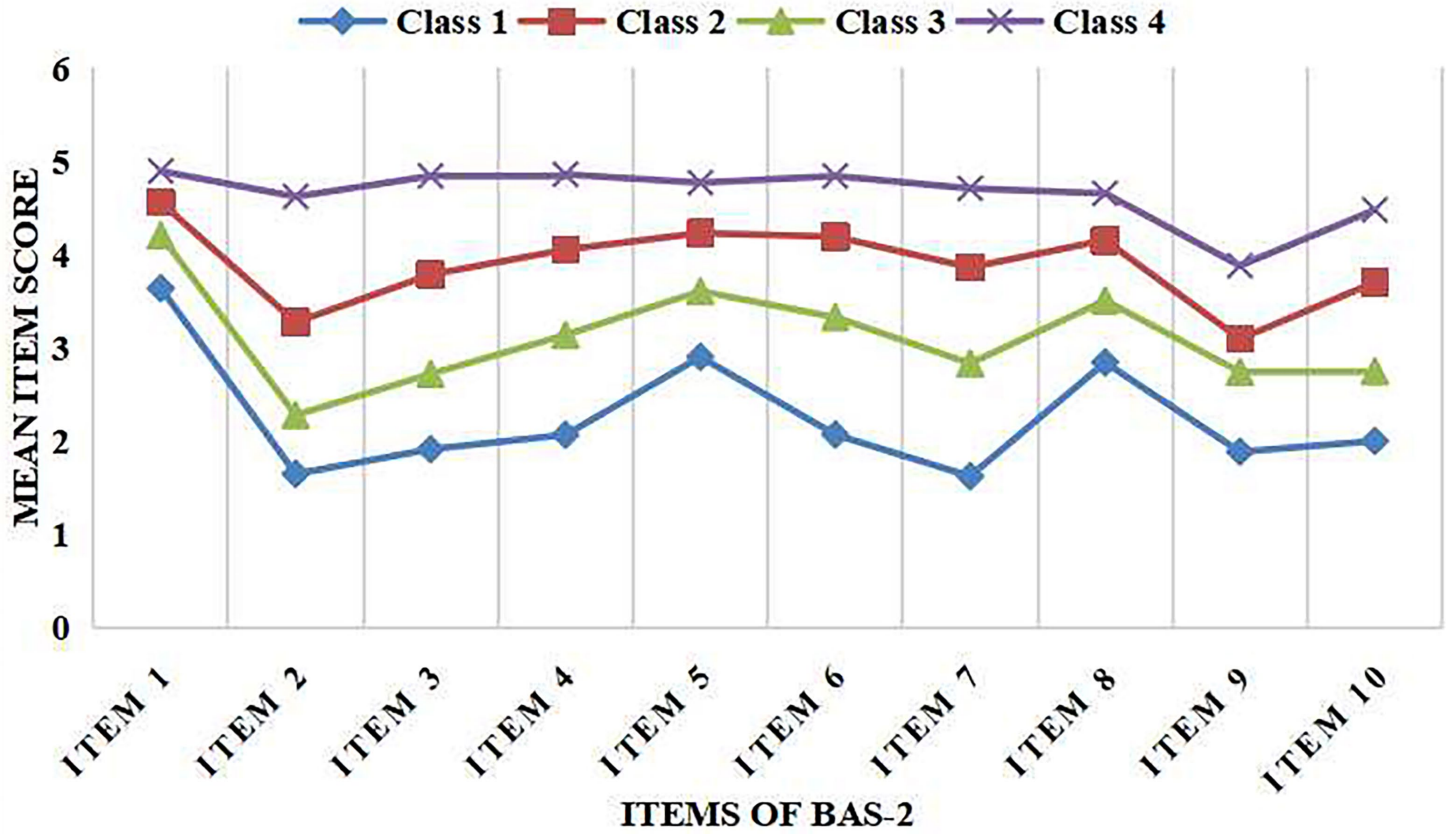
Item means for the fourth profile model of body appreciation.

We constructed seven models to explore the profiles of BA. Fit statistics are summarized in Table 3. All of the entropy values were acceptable (>0.80). However, the LMR tests were significant only for the first to fifth profiles (*p* < 0.05), eliminating the sixth and seventh profile solutions. Compared with the fifth profile model, the fourth profile model was more parsimonious and easier to interpret. Based on the parsimonious model selection criteria, the fifth profile also was rejected. Then, we applied an elbow plot to compare the fourth and third profile solutions, as shown in Figure 1. It was obvious that the value of AIC, BIC and a-BIC were significantly dropped from the first to third profile solution, and leveled off from the third profile thereafter. Consequently, the fourth profile model was deemed acceptable. Average latent class assignment probabilities for the classification were 0.925, 0.916, 0.909, and 0.901, respectively. Figure 2 shows the plot of the fourth profile model.

**Table 3:** Fit statistics for Class Models 1 through 7.

Number of profiles	AIC	BIC	a-BIC	Entropy	LMR-LRT	BLRT	Class proportions (%)
1	16137.781	16223.650	16160.162	–	–	–	100
2	14555.576	14688.672	14590.267	0.881	0.0000	0.0000	45.84/54.16
3	14137.962	14318.285	14184.962	0.854	0.0058	0.0000	19.78/45.84/34.38
4	13967.641	14195.192	14026.951	0.848	0.0221	0.0000	14.42/36.41/36.97/12.20
5	13772.997	14047.776	13844.616	0.873	0.0108	0.0000	12.75/25.51/11.09/12.38/38.26
6	13672.133	13994.139	13756.062	0.880	0.3847	0.0000	3.88/9.98/11.65/24.21/37.89/12.38
7	13610.554	13979.788	13706.792	0.854	0.4648	0.0000	2.40/15.53/10.17/10.91/16.45/11.28/33.27

AIC, Akaike information criterion; BIC, Bayesian information criterion; a-BIC: adjusted BIC; LMR-LRT, Lo–Mendell–Rubin likelihood ratio test; BLRT, bootstrap likelihood ratio test.

As illustrated in Figure 2, Class 1 (14.42% of the total sample) and Class 4 (12.20%) had the lowest and highest BA scores across the BAS-2, and were thus defined as the “low BA” group and “high BA” group, respectively. Class 2 (36.41% of the total sample) and Class 3 (36.97%), in which the BA scores were intermediate across the BAS-2, were labelled as the “upper medium BA” group and “lower medium BA” group, respectively. The total BA scores were significantly different among the four profiles (*F* = 1198.672, *p* = 0.000).

### Univariate analysis

Table 1 summarizes the results of the Chi-squared tests. There were significant differences between the four profiles of BA in years of schooling (χ^2^ = 19.799, *p* = 0.019), academic achievement level (χ^2^ = 14.100, *p* = 0.029), interpersonal relationship status (χ^2^ = 23.240, *p* = 0.000), and BMI (χ^2^ = 12.748, *p* = 0.047).

### Multinomial logistic regression analysis

The result of R3STEP tests are shown in Table 4. Years of schooling, interpersonal relationship status, BMI, and SCC were predictors of the four profiles of BA (*p* < 0.05). Specifically, compared with “high BA” group, those who were poor at interpersonal relationship status were more likely to be in the “low BA” group (B = 0.999, SE = 0.313, *p* = 0.001) or the “upper medium BA” group (B = 0.918, SE = 0.275, *p* = 0.001). Those with higher grades had higher odds of being in the “lower medium BA” group (B = 0.357, SE = 0.156, *p* = 0.022), as compared to the “high BA” group. Relative to the “high BA” group, those with higher BMI had higher odds of being in the “low BA” group (B = 0.578, SE = 0.284, *p* = 0.042). Those with lower SCC scores had higher odds of being in the “low BA” group (B = −0.087, SE = 0.023, *p* = 0.000) and “upper medium BA” group (B = −0.059, SE = 0.020, *p* = 0.003) than the “high BA” class.

**Table 4:** Predictors of four profiles of body appreciation.

Predictors	Reference Class 4								
	Class 1			Class 2			Class 3		
	*B*	SE	*p*	*B*	SE	*p*	*B*	SE	*p*
Year of schooling	0.219	0.172	0.205	0.191	0.151	0.205	0.357	0.156	0.022
Academic achievement level	0.277	0.311	0.373	0.223	0.235	0.342	−0.161	0.244	0.509
Interpersonal relationship status	0.999	0.313	0.001	0.918	0.275	0.001	0.328	0.295	0.265
BMI	0.578	0.284	0.042	0.166	0.250	0.507	−0.028	0.244	0.908
SCC	−0.087	0.023	0.000	−0.059	0.020	0.003	−0.015	0.022	0.493

Class 1, “low BA” group; Class 2, “upper medium BA” group; Class 3, “lower medium BA” group; Class 4, “high BA” group.

According to the results of BCH tests, the four profiles differed completely in the stress (**χ**^**2**^ **=** 15.206, *p* < 0.01), anxiety (**χ**^**2**^ **=** 10.999, *p* < 0.05), and depression (**χ**^**2**^ **=** 16.679, *p* < 0.01). Specifically, the “low BA” group and the “lower medium BA” group were differentiated in the stress (**χ**^**2**^ **=** 7.707, *p* < 0.01), anxiety (**χ**^**2**^ **=** 5.454, *p* < 0.05), and depression (**χ**^**2**^ **=** 5.016, *p* < 0.05); the “low BA” group and the “high BA” group were differentiated in the stress (**χ**^**2**^ **=** 10.440, *p* < 0.01), anxiety (**χ**^**2**^ **=** 7.313, *p* < 0.05), and depression (**χ**^**2**^ **=** 11.115, *p* < 0.05); with the lowest BA group reporting the highest level of negative affect. The “upper medium BA” group and the “high BA” group were differentiated by stress (**χ**^**2**^ **=** 6.602, *p* < 0.05), anxiety (**χ**^**2**^ **=** 4.999, *p* < 0.05), and depression (**χ**^**2**^ **=** 10.280, *p* < 0.01).

## Discussion

The data from our person-centered approach to evaluating BA among Chinese nursing students indicated that more than half of the students surveyed had unsatisfactory BA. This result is inconsistent with several variable-centered methods of national and international studies, which posited above average levels of BA among Chinese college students, Norwegian exercise science university students, and Omani university students, reflecting the population homogeneity (Khalaf et al., 2021; Sundgot-Borgen et al., 2021; Liu et al., 2022). As cited previously, the majority of nursing students are female, which may explain why the results differed between the general sample of college students and the nursing students (Jiang et al., 2019). Moreover, the scores of “low BA” group and “lower medium BA” group on items 1, 5, and 8 of the BAS-2 were higher than other items, indicating personal respect for one’s body. It is likely that nursing students with low and lower medium BA nonetheless respected their BI and had a positive attitude towards their appearance. This may be due to the traditional Chinese cultural value that our bodies—to every hair and bit of skin—are received from our parents, and we must not presume to injure or wound them (Stojcic et al., 2020). Meanwhile, the scores of “upper medium BA” group and “high BA” group on items 2 and 9 were lower than other items, indicating less acceptance of an individual’ s body. Students with higher levels of BA scored relatively lower on acceptance of body than on other items of the BAS-2. They were dissatisfied with their present appearance and tried to make changes in pursuit of a better appearance. This may be influenced by professional attitudes that a good nurse might not have a beautiful appearance, but it is more important to provide professional care to patients (Wainwright, 2000). Many clinical nurses prioritize the health of patients before their own (Speroni et al., 2013). For example, in an American study (Speroni et al., 2013) 72% of sample nurses were overweight or obese. Despite this statistic, the study showed that nurses were being conscious about their own BI and trying to change health habits to promote healthier lifestyles and improve appearance (Speroni et al., 2013).

The present study found that students with higher BMI had greater odds of being in the “low BA” group than in the “high BA” group. Previous variable-centered studies found that the negative relationship between BA and BMI is weak among males, and slightly greater among women. This consistency in results may be explained by the tendency to stigmatize obesity in Chinese traditional cultural context (Liu et al., 2022). However, the gender difference was insignificant on classification of BA in this study, which was inconsistent with the results of many previous variable-centered studies (Liu et al., 2022). Recently, a meta-analysis revealed that the effect size of gender differences on BA might be small, and the survey method and type of sample contributed to the heterogeneity (He et al., 2020). We finished the survey by conducting face-to-face paper questionnaires, which might have affected students’ responses to sensitive items in the BAS-2.

The present study revealed that students who were poor at interpersonal relationships were more likely to be in the “low BA” and “upper medium BA” group when compared with “high BA” group. It has been proven that social–emotional supports provided by family members and peers can influence individual BI profoundly (Seo et al., 2020). One study showed that weight-related teasing from friends and family members was an especially important factor affecting BI in youth (Seo et al., 2020). In general, parental factors have lasting effects on individual BI across the lifespan, and positive parental supports have been shown to facilitate positive BI (Seo et al., 2020). Similarly, people who get along well with peers would share their opinions with each other, and would receive reinforcement from friends about positive aspects of their appearance (Seo et al., 2020). Purportedly the most influential source of BI for youth is social media. Social media can influence self-cognition when young people compare themselves with idealized BI and try to change their appearance in pursuit of an unrealistic goal (Rounsefell et al., 2020). In China, middle school students have fewer opportunities to use social media compared with college students. College students in higher grades might be more seriously affected by social media, and in our study, they had higher odds of being in the “lower medium BA” group, as compared to the “high BA” group.

This study revealed that SCC was a predictor of classification of BA among Chinese nursing students. Scholars have contended that lower SCC contributes to lower self-esteem and lower self-control (Rahim et al., 2021). In addition, SCC has been considered to be a dispositional tendency, varying across one’ s lifespan (Rahim et al., 2021). Because the ideals and beliefs of many college students are changeable, their self-views are more likely to be influenced by factors in their social environment (Na et al., 2018). When experiencing stressors, students with higher SCC can evaluate situations and be more resilient in coping with perceived stress (Lee, 2021). In contrast, students with lower SCC are easily affected by negative factors and are more likely to perceive them as threats (Lee, 2021). Many undergraduate students, especially those with low SCC, have changeable self-views (Na et al., 2018). As mentioned previously, family members, peers, and social media are the most important sources of body dissatisfaction for youth, and these relationships are often mediated by social comparison or thin-ideal internalization. Students with high SCC are less influenced by comparison with unrealistic ideals of attractiveness (Vartanian and Dey, 2013). Conversely, students with low SCC usually experience body dissatisfaction when comparing themselves to unattainable beauty ideals (Vartanian and Dey, 2013). It is likely that programs targeting development of a clear self-concept may help to improve BA by decreasing body-image-related social comparison or thin-ideal internalization.

The four BA profiles that we analyzed differed in stress, depression, and anxiety among the students, which was consistent with variable-centered studies. Recently, a Chinese nationwide survey proved the negative relationship between BA and psychiatric symptoms of depression, anxiety, and suicidality among samples of all college students and female students (Liu et al., 2022). Although, nursing faculties tend to concentrate on academic-related negative affect, body dissatisfaction is a non-negligible risk factor of several psychiatric symptoms (Tung et al., 2018; Liu et al., 2022).

Considering the many factors affecting classification of BA that this study highlighted, BI should be an important consideration in nursing curricula. Nursing care is human art in which nurses apply their prior knowledge and skills (Siles-González and Solano-Ruiz, 2016). In addition, it is important to help students form a positive understanding of the impact of BI and esthetics on nurses and the care that they provide (Atkins et al., 2018). An approach that combines both science and art can create more pleasant experiences in the daily work of caring for patients (Siles-González and Solano-Ruiz, 2016). Beyond the work of caring itself, what we communicate to patients with looks, words, and feelings creates an esthetic that should be positive and pleasant (Siles-González and Solano-Ruiz, 2016). Moreover, nurses’ positive attitudes toward health and BI may lead patients to adopt a healthy lifestyle (Atkins et al., 2018). Consequently, nurses and nursing students should take care of themselves first to professionally and optimally meet the needs of patients. It is important for nursing students to develop healthy self-care habits, including exercise, eating healthy, maintaining optimum BMI, and establishing a healthy network of supportive relationships. Besides, interventions that are effective in promoting SCC may be helpful in enhancing the development of BA. Because SCC is associated with sense of autonomy, nursing faculties can help nursing students pursue their own values of life, sense of meaning, and spiritual ideals and to integrate those ideals into identifying themselves (Rahim et al., 2021). This may contribute to the formation of positive evaluations about themselves, which in turn can result in facilitating the development of BA.

This study had several limitations. First, because of the shortage of human and material resources, we conducted the survey with a limited sample size in only one nursing college, using convenience sampling. Future research might use a larger, more representative, multi-site sample. Second, the study was a cross-sectional survey, so we were unable to establish causal relationships between SCC, body appreciation, and negative affect among sample students. Future research might use a longitudinal study design to confirm our findings. Finally, we examined a limited number of predictive factors of BA for Chinese nursing students. More predictive factors (e.g., resilience, body image comparison of social networking sites, family atmosphere, problematic social media use, social communication skills) should be considered in future research.

## Conclusion

From a person-centred perspective, this study highlighted the heterogeneity of BI among the sample, but results indicated that more than half of nursing students were unsatisfied with their bodies. Moreover, we identified the predictive factors (years of schooling, interpersonal relationship status, BMI, SCC) of profiles of BA and its associations with stress, depression, and anxiety among Chinese nursing students. This has implications for nursing education. Nursing faculties should provide guidance on appearance, especially for obese students with high grades and poor interpersonal relationship status. It is important to evaluate the role of BI in psychiatric symptoms such as anxiety and depression. Further, we should encourage students to learn the aesthetics of nursing as well as the science, to maintain a healthy lifestyle, and to improve negative self-esteem through repair of parental and peer relationships. Additionally, while considering the heterogeneity of nursing students, faculties should develop programs that guide students in forming a clearer self-concept to decrease the effects of social environmental factors. By focusing appropriately on appearance, and reducing body-image-related social comparisons and internalization of unrealistic ideals, students can improve their appreciation of their bodies.

## Data availability statement

The raw data supporting the conclusions of this article will be made available by the authors, without undue reservation.

## Ethics statement

The studies involving human participants were reviewed and approved by the Ethics Committee of the Anhui Medical University (20210075). Written informed consent to participate in this study was provided by the participants’ legal guardian/next of kin.

## Author contributions

TZ wrote and revised the manuscript. HL and DS designed the study. SL and YY collected and analyzed the data. All authors contributed to the article and approved the submitted version.

## Funding

This work was supported by the University Natural Science Research Project of Anhui Province (approval number KJ2021A0256, KJ2020A0221), Education Quality Engineering Project of Anhui Province (approval number 2020kcszyjxm124), and Natural Science Research Project of School of Nursing of Anhui Medical University (approval number hlpy20210019).

## Conflict of interest

The authors declare that the research was conducted in the absence of any commercial or financial relationships that could be construed as a potential conflict of interest.

## Publisher’s note

All claims expressed in this article are solely those of the authors and do not necessarily represent those of their affiliated organizations, or those of the publisher, the editors and the reviewers. Any product that may be evaluated in this article, or claim that may be made by its manufacturer, is not guaranteed or endorsed by the publisher.
